# Oral Transmission of Chagas Disease by Consumption of Açaí Palm Fruit, Brazil

**DOI:** 10.3201/eid1504.081450

**Published:** 2009-04

**Authors:** Aglaêr A. Nóbrega, Marcio H. Garcia, Erica Tatto, Marcos T. Obara, Elenild Costa, Jeremy Sobel, Wildo N. Araujo

**Affiliations:** Brazilian Ministry of Health, Brasília, Brazil (A.A. Nóbrega, M.H. Garcia, E. Tatto, M.T. Obara, W.N. Araujo); Secretariat of Public Health, Belem, Brazil (E. Costa); Centers for Disease Control and Prevention, Atlanta, Georgia, USA (J. Sobel); Gonçalo Muniz Institute, Salvador, Brazil (W.N. Araujo)

**Keywords:** Acute Chagas disease, foodborne disease, açaí palm fruit, oral transmission, Amazon, Brazil, dispatch

## Abstract

In 2006, a total of 178 cases of acute Chagas disease were reported from the Amazonian state of Pará, Brazil. Eleven occurred in Barcarena and were confirmed by visualization of parasites on blood smears. Using cohort and case–control studies, we implicated oral transmission by consumption of açaí palm fruit.

Chagas disease (American trypanosomiasis) chronically infects ≈10 million persons in Latin America (*1*). The etiologic agent is *Trypanosoma cruzi*, which is transmitted by bloodsucking triatomine insects. Other modes of transmission are transfusional, congenital, and oral (foodborne) (*2*). Oral transmission occurs by consumption of foods contaminated with triatomines or their feces or by consumption of raw meat from infected mammalian sylvatic hosts (*3*). The precise stage of food handling at which contamination occurs is unknown. The first outbreak of orally transmitted Chagas disease in Brazil was reported in 1965 (*4*). Two outbreaks were associated with consumption of sugar cane juice (*5**,**6*). In these outbreaks, the incubation period was ≈22 days, compared with 4–15 days for vectorial transmission and 30–40 days for transfusional transmission (*7*).

Chagas disease has not been considered endemic in the Brazilian Amazon region. The first Amazonian outbreak of acute Chagas disease was reported in 1968; oral transmission was suspected (*8*). During 1968–2005, a total of 437 cases of acute Chagas disease were reported in this region. Of these cases, 311 were related to 62 outbreaks in which the suspected mode of transmission was consumption of açaí (*9*).

Açaí is the fruit of a palm of the family *Aracaceae* (Figure 1, panel A); it is crushed to produce a paste or beverage. Most of the Amazonian population consumes açaí juice daily. Contamination is believed to be caused by triatomine stools on the fruit or insects inadvertently crushed during processing (*10*). There are no reports of collection of açaí for laboratory testing during an outbreak of acute Chagas disease. Because outbreaks with high attack rates occur in small groups whose members all consume the same foods, açaí has not been epidemiologically implicated in transmission of this disease.

**Figure 1:**
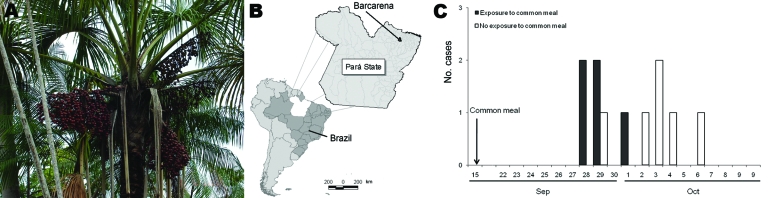
A) Açaí palm and açaí fruit. B) Location of Barcarena in Pará State, Brazil. C) Epidemic curve for 11 case-patients with acute Chagas disease, Barcarena, Brazil, September–October 2006.

During January–November 2006, a total of 178 cases of acute Chagas disease were reported in Pará State, Brazil, in the Amazon basin (Ministry of Health, unpub. data). Eleven of these cases occurred in Barcarena (population 63,268) (*11*) (Figure 1, panel B). All patients had symptom onset in September and October. Of the 11 case-patients, 5 were staff members at a health post who shared a meal at a staff meeting on September 15. We attempted to identify risk factors for illness.

## The Study

We conducted a retrospective cohort study of staff members at the health post who participated in the meeting on September 15. A case-patient was any person who participated in the meeting and had a positive direct parasitologic examination result for *T*. *cruzi* or positive serologic results and clinical evidence of acute Chagas disease. A non-case was any person who participated in the meeting and had negative test results for *T*. *cruzi*. We also conducted a 1:3 case–control study (11 case-patients and 34 controls matched by sex and age) that included patients with laboratory-confirmed cases from Barcarena. A case-patient was any person in whom during September 1–October 15 *T*. *cruzi* was found by direct parasitologic examination, irrespective of signs or symptoms of disease, or who had positive serologic results and clinical evidence of disease. This interval was based on date of symptom onset of the first and last case-patient and a reported incubation period of 3–22 days for orally transmitted disease. Controls were age- and sex-matched residents of case-patient neighborhoods who had negative serologic results for *T*. *cruzi*.

Parasitologic examinations were conducted for case-patients by using quantitative buffy coat test, thick blood smear, or buffy coat test (the latter 2 tests included Giemsa staining). Serologic tests were conducted by using indirect hemagglutination test, ELISA, or indirect immunofluorescent test. An immunoglobulin (Ig) M titer >40 was considered positive. Controls had nonreactive IgM and IgG titers. We ruled out leishmaniasis in all persons with positive serologic results for *T*. *cruzi* by using an immunofluorescent test for IgM to *Leishmania* spp. (*12*).

We conducted an entomologic investigation during December 11–16, 2006, at the homes of 5 case-patients and in forested areas near the homes of 2 case-patients; at the commercial establishment where açaí consumed by the case-patients linked to the health post was prepared and served; at an açaí juice production and sale establishment reported to be frequented by other case-patients; and at the river dock market where açaí delivered to Barcarena is unloaded. At this market, we searched baskets used to transport açaí in river boats. We applied an insect-displacing compound (piridine; Pirisa, Taquara, Brazil) to the interior and exterior of buildings at investigation sites and placed traps (*13*) to obtain triatomines.

Data were analyzed by using Epi Info version 6.04d (Centers for Disease Control and Prevention, Atlanta, GA, USA). We measured relative risk in the cohort study and matched odds ratios in the matched case–control study, with 95% confidence intervals and α = 5%. Fisher exact, McNemar, Mantel-Haenszel, and Kruskall-Wallis tests were used as needed. Study power (1 – β) was 5%.

**Figure 2:**
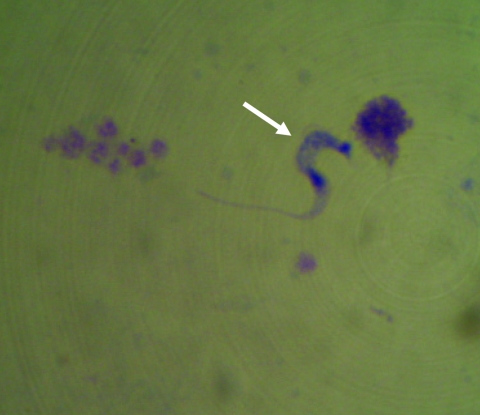
*Trypanosoma cruzi* (arrow) in a peripheral blood smear of a patient at a local health facility in a rural area of Pará State, Brazil (Giemsa stain, magnification ×100). Image provided by Adriana A. Oliveira, Brazilian Field Epidemiology Training Program, Brasilia, Brazil.

All case-patients had positive results for *T*. *cruzi* by direct examination of blood (Figure 2). Nine (82%) patients were female; median age was 39 years (range 7–70 years). Eight (73%) patients resided in urban areas, 7 (64%) in brick dwellings, and 3 (27%) in mixed brick and wooden dwellings. All patients denied having had blood transfusions or organ transplants, having slept in rural or sylvatic areas, and having been bitten by triatomines.

The epidemic curve for the 11 patients is shown in Figure 1, panel C. Main signs and symptoms were fever, weakness, facial edema, myalgia, arthralgia, and peripheral edema (Table 1). No deaths occurred, and median time from symptom onset to treatment initiation was 22 days.

**Table 1:** Signs and symptoms in 11 patients with laboratory-confirmed acute Chagas disease, Barcarena, Brazil, 2006

Sign or symptom	No. (%) patients
Fever	11 (100)
Fatigue	11 (100)
Facial edema	11 (100)
Headache	10 (91)
Myalgia	9 (82)
Arthralgia	9 (82)
Peripheral edema	9 (82)
Shortness of breath	7 (64)
Tachycardia	7 (64)
Nausea/vomiting	7 (64)
Jaundice	5 (46)
Epigastric pain	5 (46)
Retroorbital pain	5 (46)

The cohort consisted of 12 persons who attended the staff meeting. Of these persons, 6 shared a meal, 5 (83%) of whom were case-patients. The remaining persons were seronegative for *T*. *cruzi*. Exposures associated with infection were consumption of thick açaí paste and drinking açaí juice at the health post; consumption of chilled açaí was protective (Table 2). This shared meal was the only common exposure among cohort members. No other foods consumed at the meal were associated with illness (Table 2). Among exposures tested, drinking açaí juice on September 15 and at the health post were significantly associated with illness (p<0.02 and p<0.001, respectively; matched odds ratio not determined). Other exposures were not associated with illness. No triatomine insects were identified at any sites of the entomologic investigation.

**Table 2:** Food exposures in a cohort study of 5 case-patients with acute Chagas disease, Barcarena, Brazil, 2006*

Exposure†	Ill, no. (%)	Not ill, no. (%)	RR	95% CI	p value‡
Açaí, thick paste	3 (100)	0	4.5	1.3–15.3	0.04
Açaí juice at health post	3 (100)	0	4.5	1.3–15.3	0.04
Chilled açaí juice	1 (12)	7 (88)	0.1	0.02–0.8	0.02
Charque	3 (75)	2 (25)	5.3	0.8–35.1	0.09
Cupuaçu	2 (100)	0	3.3	1.3–8.6	0.15
Biribá	1 (50)	1 (50)	1.3	0.3–6.1	0.68
Muruci	1 (100)	0	2.3	1.3–6.0	0.42
Any raw food	4 (67)	2 (33)	4.0	0.6–26.1	0.12

*RR, relative risk; CI, confidence interval. †Charque is dried, salted meat; cupuaçu, biribá, and muruci are fruits. ‡By Fisher exact test.

## Conclusions

Our study findings implicated açaí in an outbreak of acute Chagas disease. Oral transmission of this disease in the Amazon region has been reported since the 1960s. Açaí has long been the principal suspected food vehicle, but characteristics of outbreaks, small groups with universal exposure and high attack rates, have precluded epidemiologic implication of this food. There are no reports of timely collection of açaí for laboratory testing in an outbreak.

In this outbreak, vectorborne, transfusional, transplant-associated, and transplacental transmission were excluded. Incubation periods of cohort case-patients were compatible with those of previous reports. A shared meal was the only event linking case-patients, and cohort and case–control studies demonstrated an association between açaí consumption at this meal and infection. These findings indicate an outbreak of orally transmitted disease from contaminated açaí.

Limitations of this study are possible recall bias caused by delay between illness and investigation and failure to collect food samples for testing. Studies are needed to determine viability of *T*. *cruzi* in açaí, along with the tree-to-bowl continuum of açaí, to identify sources of contamination. Because açaí is a major dietary component in the Amazon region and a component of the local economy, identifying practical prevention measures is essential.
